# eIF5B regulates the expression of PD-L1 in prostate cancer cells by interacting with Wig1

**DOI:** 10.1186/s12885-021-08749-w

**Published:** 2021-09-15

**Authors:** Qi Li, Mulun Xiao, Yibo Shi, Jinhao Hu, Tianxiang Bi, Chaoliang Wang, Liang Yan, Xiaoyan Li

**Affiliations:** 1 Department of Urology, The First Affiliated Hospital of Zhengzhou University, Zhengzhou City, 450052 Henan Province China; 2 Department of Neonatel Intensive Care Unit, Zhengzhou First People’s Hospital, Zhengzhou City, 450004 Henan Province China

**Keywords:** eIF5B, Wig1, PD-L1, Prostate cancer

## Abstract

**Background:**

Eukaryotic translation initiation factors (eIFs) are the key factors to synthesize translation initiation complexes during the synthesis of eukaryotic proteins. Besides, eIFs are especially important in regulating the immune function of tumor cells. However, the effect mechanism of eIFs in prostate cancer remains to be studied, which is precisely the purpose of this study.

**Methods:**

In this study, three groups of prostate cancer cells were investigated. One group had its eIF5B gene knocked down; another group had its Programmed death 1 (PD-L1) overexpressed; the final group had its Wild-type p53-induced gene 1 (Wig1) overexpressed. Genetic alterations of the cancer cells were performed by plasmid transfection. The expression of PD-L1 mRNA was detected by quantitative real-time PCR **(**qRT-PCR), and the expressions of PD-L1 and eIF5B proteins were observed by western blot assays. Cell Counting Kit-8 (CCK-8), flow cytometry, Transwell and Transwell martrigel were used to investigated cell proliferation, apoptosis, migration and invasion, respectively. The effect of peripheral blood mononuclear cells (PBMCs) on tumor cells was observed, and the interaction between eIF5B and Wig1 was revealed by co-immunoprecipitation (CoIP) assay. Finally, the effects of interference with eIF5B expression on the growth, morphology, and immunity of the tumor, as well as PD-L1 expression in the tumor, were verified by tumor xenograft assays in vivo.

**Results:**

Compared with normal prostate epithelial cells, prostate cancer cells revealed higher expressions of eIF5B and PD-L1 interference with eIF-5B expression can inhibit the proliferation, migration, invasion and PD-L1 expression of prostate cancer cells. Meanwhile, the cancer cell group with interference with eIF5B expression also demonstrated greater, apoptosis and higher vulnerability to PBMCs. CoIP assays showed that Wig1 could bind to eIF5B in prostate cancer cells, and its overexpression can inhibit the proliferation, migration, invasion and PD-L1 expression of cancer cells while promoting apoptosis. Moreover, interference with eIF5B expression can inhibit tumor growth, destroy tumor morphology, and suppress the proliferation of tumor cells.

**Conclusion:**

eIF5B can promote the expression of PD-L1 by interacting with Wig1. Besides, interference with eIF5B expression can inhibit the proliferation, migration, invasion and immunosuppressive response of prostate cancer cells. This study proposes a new target, eIF5B, for immunotherapy of prostate cancer.

**Supplementary Information:**

The online version contains supplementary material available at 10.1186/s12885-021-08749-w.

## Introduction

Prostate cancer is one of the most common cancers in the male genitourinary system, and its incidence rate increases as people age. Moreover, its global incidence rate ranks second among all malignant tumors in males, and its mortality rate is the fifth of all malignant tumors [1]. Previous studies have shown that prostate cancer is a low immune-reactive cancer characterized by limited infiltration of immune cells or extensive infiltration of immunosuppressive T cells, in which the processed death-1 (PD-1) /programmed death ligand 1 (PD-L1) pathway plays an important role [2]. In general, the PD-1/PD-L1 signaling pathway could inhibit the body’s immunity to tumors, and the pathway could be blocked by inhibiting PD-L1 expression, thereby enhancing immune function and killing the tumors. Although PD-1 immunotherapy has been approved as the first-line treatment for patients with prostate cancer, the mechanism of PD-L1 up regulation in prostate cancer is still unclear. Therefore, it is of great significance to identify PD-L1 regulators and relevant clinical biomarkers to predict patients’ response to immunotherapy and provide a new strategy to treat prostate cancer.

Eukaryotic translation initiation factor (eIF) is an essential factor participating in the formation of translation initiation complexes during protein synthesis in eukaryotic cells. More than 20 eIFs have been found till now, and eIF-2, eIF-3, eIF-4 and eIF-5 are the most studied subtypes. Various eIFs have been demonstrated as important participants in tumor occurrence and development, as well as the initial stage of protein translation in eukaryotic cells. Specifically, eIF-4E is the first identified eIF that is highly expressed in tumors, and its expression level is subject to the tumor stage [3, 4]. Moreover, researchers also found that many eIFs are highly expressed in tumors, and they are involved in tumor occurrence, invasion and metastasis [5, 6]. For example, eIF5B is a subunit of eIF-5 and a GTPase, and it is capable to specifically activate the GTPase enzymatic activity of eIF-2 [7]. Shruthy et al. revealed that eIF5B is overexpressed in lung adenocarcinoma, which induces PD-L1 overexpression and poor prognosis [8]. However, the effect of eIF5B on immunosuppression in prostate cancer has not been studied yet.

p53 is a tumor suppressor gene that is capable to repair damaged DNA and inhibit the activation of oncogenes, thereby inhibiting tumor development [9]. In human tumors, the mutation frequency of p53 is as high as 50% [10]. Evidence has shown that p53 and PD-L1 expressions are correlated in various malignant tumors [11, 12]. Particularly, wild-type p53-induced gene 1(Wig-1) is a target gene of p53. It encodes an unusual zinc finger protein and participates in the regulation of post-transcriptional genes. Previous studies have shown that Wig-1 can bind to both long double-stranded RNA and short dsRNA chains. Besides, Wig-1 also initiates a positive feedback loop through the gold rich element (GRE) in the untranslated region of the 3′ end in p53 to stabilize p53 mRNA. Finally, Wig-1 is highly expressed in tumor cells and may be involved in apoptosis and oxidative stress. Hyung et al. found that Wig-1 interacts with eIF5B to inhibit the initiation procedure of translation [13]. Following that, the present study will investigate whether eIF5B affects PD-L1 expression in prostate cancer cells by interacting with Wig1.

## Materials and methods

### Cell culture

RWPE-1, a normal prostatic epithelial cell line, and PC-3 and VCaP, two prostate cancer cells lines were obtained from the American Type Culture Collection (ATCC, Manassas, VA). All cells were cultured in the medium recommended by ATCC and incubated in a humidified incubator at 37 °C and 5% CO_2_. Specifically, RWPE-1 cells were cultured in MEM supplemented with 10% fetal bovine serum (FBS), and the culture medium was free of antibiotics. PC-3 and VCaP cells were cultured in an F12 medium containing 10% FBS without antibiotics.

Peripheral blood mononuclear cells (PBMCs) were isolated from white blood cells of healthy donors (Changsha blood center, Hunan Province, China) by Ficoll -Paque gradient centrifugation. Subsequently, the PBMCs were cultured in RPMI 1640 medium supplemented with 10% FBS, 50 U/ml penicillin and 50 μg/ml streptomycin in an incubator at 37 °C and 5% CO_2_.

### Cell transfection

Before transfection, PC-3 and VCaP cells in the logarithmic growth phase were seeded on several 24-well plates and cultured until the cell density reached 80%. Following the instructions of lipofectamine 2000 transfection reagent (Invitrogen, USA), NC plasmids and those with eIF5B siRNA interference were transfected in the cells. Both NC and eIF5B siRNAs were purchased from Shanghai Tuoran Biotechnology Co., Ltd.

Lentiviruses that expressed sequence shRNAs specific to eIF5B (sh-eIF5B) were designed and synthesized by Shanghai Sangong Biotechnology Co., Ltd. (China) to knockdown eIF5B in PC-3 and VCaP cells. Nontarget shRNA lentiviruses (sh-NC) were used as the negative control. Plasmids with PD-L1 and Wig1 overepressions were obtained from GeneChem Company (China), and the plasmids were then transfected to the two cell lines using Lipofectamine 2000 (Invitrogen, USA) following the manufacturer’s protocol. Subsequently, cells transfected with lentiviruses were selected by puromycin (11,000, Solarbio, China) for at least 48 h and thereafter cultured normally.

### Cell proliferation assay

Cell Counting Kit-8 (CCK-8) kit (Boster Biological, China) was used to detect cells proliferation following the manufacturer’s instructions. Briefly, cells were seeded into several 96-well plates with a cell density of 5 × 10^3^ cells per well and cultured for 72 h. Subsequently, 10 μl CCK-8 solution was added to each well, and the cells were incubated for another 1 h. Finally, the cells’ absorbance at 450 nm was measured by a microplate reader.

### Apoptosis analysis

The percentage of apoptotic cells was measured 72 h after transfection, and the measurements were performed using the Annexin V-fluorescein isothiocyanate (FITC)/propidium iodide (PI) double staining protocol following the manufacturer’s instructions. The cells were treated with 0.25% trypsin for 24 h and rinsed with PBS 3 times before being resuspended in 300 μL of binding buffer. Finally, Annexin V-FITC/PI was added to the suspension. Cell apoptosis was observed by flow cytometry and analyzed by Cell Quest.

### Cell migration and invasion assay

Cell migration and invasion were detected using Transwell chambers (Costar, USA). For the migration assay, transfected cells in the logarithmic growth phase were treated with trypsin and resuspended into individual cell suspensions, in which the cell density was adjusted to 1.5 × 10^6^ cells/ml. The cell suspensions were added into the Transwell upper chamber at an amount of 200 μl/well, and 600 μl of complete medium was added into the Transwell lower chamber. For the invasion assay, Matrigel glue (BD Biosciences, USA) was administered evenly on the inner membrane of Transwell chambers and air-dried before use. The cell density in the chambers was adjusted to 1 × 10^6^ cells/ml, and a recipe of solutions that is identical to that in the migration assay was applied in both chambers. Cells were incubated for 12 h and 24 h for migration and invasion assays, respectively. After incubation, the cells were fixed with formaldehyde for 15 min, stained with crystal violet for another15 min, and inspected with an optical microscope (× 100). Four visual fields were randomly selected and had the number of cells within counted. The experiment was performed in triplicates.

### Quantitative real-time PCR (qRT-PCR)

Total RNA was extracted with Trizol (Takara, Japan), and the cDNA was reverse-transcribed from the total RNA by an RNA reverse transcription kit (Applied Biosystems, USA). An SYBR Green RT-PCR kit (Takara, Japan) and an ABI prism 7500 instrument (Applied Biosystems, USA) were used for RT-PCR, and the reaction conditions were as follows: pre-denaturation at 95 °C for 3 min, 40 cycles (95 °C 30 s, 60 °C 45 s), and extension at 72 °C for 6 min. The primers were synthesized by Shanghai Sangong Biotechnology Co., Ltd. (China), and they are summarized below:

PD-L1 forward: 5′- TGCGGACTACAAGCGAATCA-3′; PD-L1 reverse: 5′- GATCCACGGAAATTCTCTGGTT-3′; GAPDH (internal control) forward: 5′-CCAGGTGGTCTCCTCTGA-3′; GAPDH (internal control) reverse: 5′-GCTGTAGCCAAATCGTTGT-3′. The relative gene expression levels were calculated by the 2^-△△Ct^ method.

### Western blot assays

Cells and tissues were collected and lysed with RIPA lysis buffer (Thermo Fisher Scientific, USA). After centrifugation, the lysate was collected and detected for its protein concentration by a BCA kit (Thermo Fisher Scientific, USA). Following an SDS-PAGE electrophoresis session on the lysate, the proteins obtained were transferred to several PVDF membranes (Millipore, USA), and the membranes were blocked in 5% BSA for 1 h before being incubated with primary antibodies at 4 °C overnight. Anti-PD-L1 and anti-GAPDH antibodies were purchased from Abcam (USA) and used following the manufacturer’s protocol. The PVDF rinsed with TBST three times and membranes were incubated with an HRP-conjugated secondary antibody (Santa Cruz, USA) for 1 h at room temperature. Then, the membranes were treated with an ECL kit (Thermo Fisher Scientific), and images were acquired using the ChemiDoc Imaging system.

### Measurement of PBMCs-induced tumor cell-killing

The prostate cancer cells transfected with NC plasmids or those with eIF-5B siRNA interference were seeded into several 96-well plates at a cell density of 5 × 10^3^ cells/well and cultured overnight at 37 °C and 5% CO2. On the next day, the cancer cells were incubated with PBMCs at an effect-target ratio of 5:1 for 48 h. The supernatants in each group were collected for co-incubation, andlactate dehydrogenase (LDH) secretions were observed by an ELISA kit (Abcam, USA) following the manufacturer’s instructions. Meanwhile, lymphocytes in each culture were collected and counted under a light microscope. The apoptosis of CD4+ T and CD8+ T lymphocytes, as well as PD-L1 expression, were detected by flow cytometry.

### Co-immunoprecipitation (CoIP) assay

48 h after the transfection, the cells were rinsed twice with PBS and lysed in lysis buffer (20 mm/L Tris HCl, 1% np40, 150 mmol/L NaCl, 10% glycerol, and a mixture of protease inhibitors) for 30 min. The lysate was centrifuged at 15000 g and 4 °C for 10 min, and the supernatant was cleared with Protein G Agarose beads. Subsequently, the target protein antibodies were added for immunoprecipitation, and the precipitated materials were analyzed by SDS-PAGE and Western blot.

### Tumor xenograft assays

All animal procedures were approved by the Ethics Committee of the First Affiliated Hospital of Zhengzhou University (Ethics number: Swearton (F) No.2020177). 5-week-old male BALB/C nude mice were purchased from Beijing Weitong Lihua Experimental Animal Technology Co., Ltd., and all mice weighed 20–25 g. Prostate cancer cells transfected with sh-control or sh-eIF5B were made into cell suspensions at a concentration of 5 × 10^7^ cells/ml. Under sterile conditions, 0.1 ml of the cell suspensions above was subcutaneously injected into the right back of each nude mouse with disposable syringes. The longest diameter (L), longest transverse diameter perpendicular to the longest diameter (W) and the height (H) of the tumor were measured with a ruler to calculate the tumor volume (V) following the equation below: V = L × W × H × π/6. 24 nude mice with V ≥ 100 mm^3^ were randomly divided into 4 groups, namely sh-control + anti-PD-L1, sh-eIF5B + anti-PD-L1 group, sh-control + IgG control, and sh-eIF5B + IgG control groups. In the first two groups, 200 μg anti-PD-L1 was injected intraperitoneally into the mice every 3 days for four times; in the latter two groups, the mice were injected with the same dose of IgG instead as the control. 35 days after tumor transplantation, the mice were euthanized by carbon dioxide asphyxiation. Following volume calculations, the tumor tissues were weighed and examined by HE staining.

### Immunostaining

Tumor tissues were embedded in paraffin and sliced into slices of 7 μm. Subsequently, the slices were fixed with methanol and permeabilized with 0.1% Triton X-100 in PBS for 20 min. Following that, the tissues were incubated with eIF5B primary antibodies overnight at 4 °C before being, incubated in fluorochrome-conjugated or normal secondary antibodies for 2 h at room temperature. A Dako LSAB detection system (catalog#k0679, DAKO USA) was used for visualization.

### Statistical analysis

SPSS 20.0 was used for statistical analyses. All data were expressed as mean ± standard deviation (SD) and analyzed using the one-way ANOVA test and Student’s t-test. Differences with a *p* < 0.05 were considered statistically significant.

## Results

### Effect of eIF5B silencing on prostate cancer cells

**Fig. 1:**
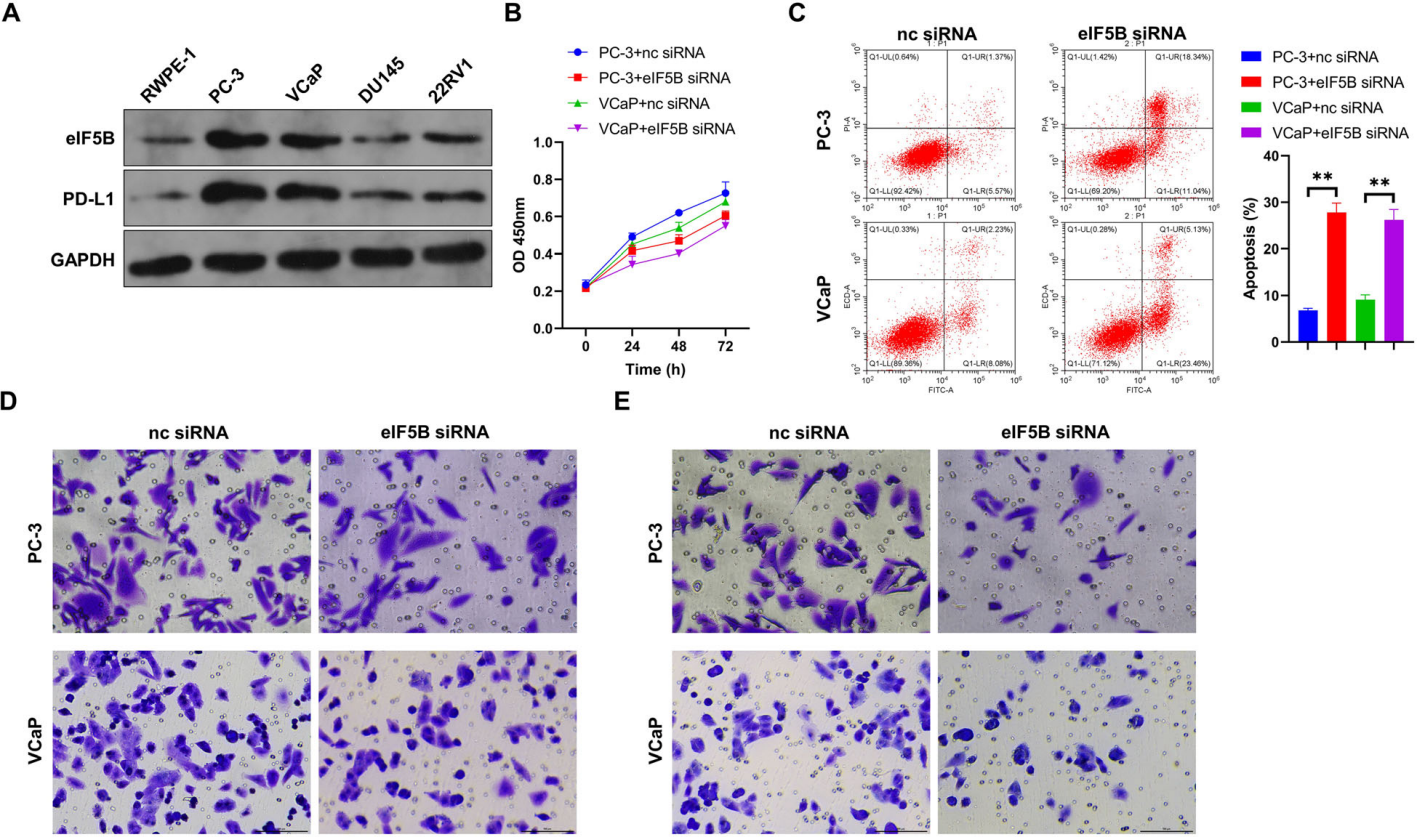
Effects of eIF5B on prostate cancer cells. A: The levels of eIF5B and PD-L1 proteins in RWPE-1, PC-3, VCaP, DU145, and 22RV1 cells (full-length blots are presented in Additional file 1); Cell proliferation (B), apoptosis (C), cell migration (D) and cell invasion (E) of prostate cancer cells transfected with si-eIF5B or si-NC; **P < 0.01

Western blot was used to detect the protein expressions of eIF5B and PD-L1 in human normal prostate epithelial cells and prostate cancer cells. According to the results, eIF5B and PD-L1 revealed significantly higher expressions in PC-3 and VCaP cells compared to RWPE-1 (Fig. 1A), so we chose PC-3 and VCaP for follow-up experiments. To verify the effect of eIF5B on prostate cancer cells, we transfected eIF5B shRNA and its control into PC-3 and VCaP. Compared to those in the NC siRNA groups, cancer cells in the eIF5B siRNA groups demonstrated significantly lower capabilities of proliferation, migration, and invasion. Besides, the latter cells also showed a significant rise in the apoptosis rates (*P*<0.01, Fig. 1B-E). In a word, our results suggest that interference with eIF5B expression could inhibit the proliferation, migration and invasion of prostate cancer cells, while promoting their apoptosis.

### eIF5B silencing inhibits PD-L1 expression in prostate cancer cells

**Fig. 2:**
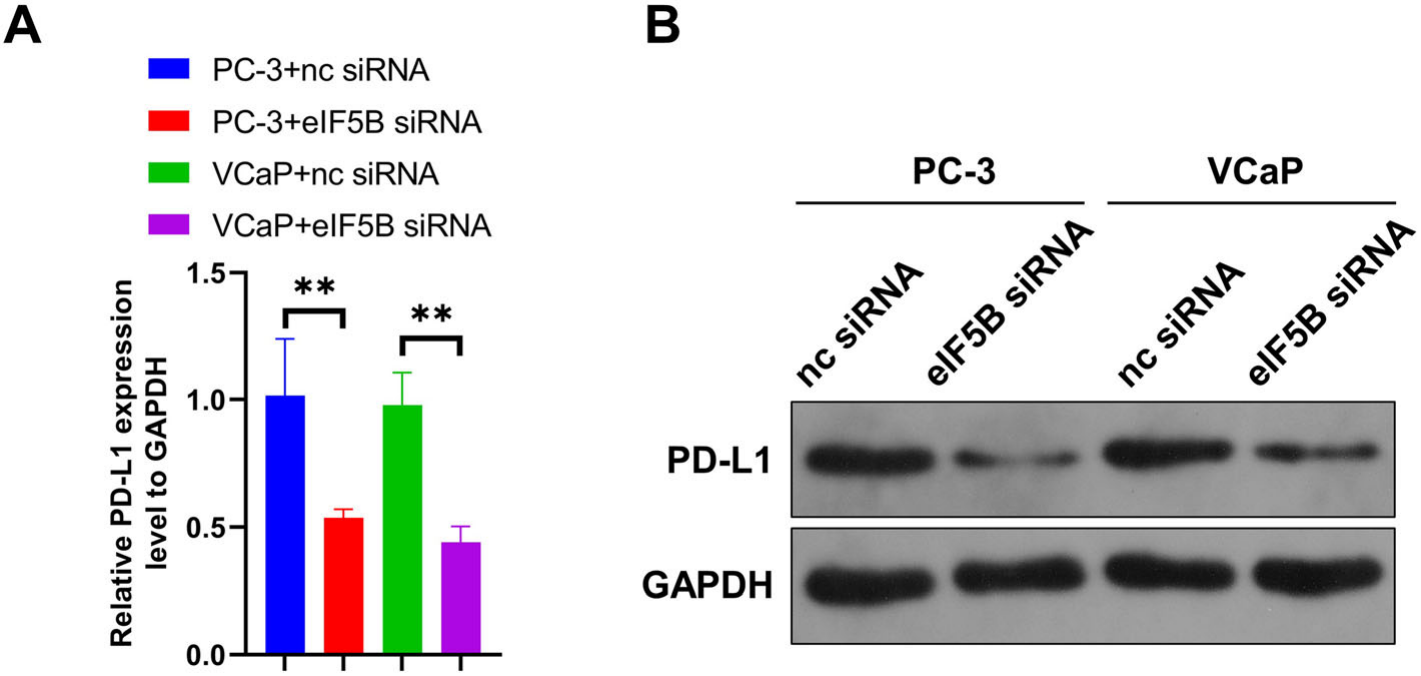
Knockdown of eIF5B inhibits the expression of PD-L1 in prostate cancer cells. The expressions of PD-L1 in prostate cancer cells were detected by qRT-PCR (A) and Western blot (full-length blots are presented in Additional file 1) (B); **P < 0.01

To explore the regulatory correlation between eIF5B and PD-L1 in prostate cancer cells, we detected the expression levels of PD-L1 mRNA and PD-L1 protein in PC-3 and VCaP cell lines after interference with eIF5B expression by qRT-PCR and Western blot. Judging from the results, the expressions of PD-L1, both its mRNA and protein, were significantly inhibited in the eIF5B siRNA groups (*P*<0.01, Fig. 2A and B), suggesting that interference with eIF5B expression could inhibit PD-L1 expression in prostate cancer cells.

### eIF5B silencing enhances the killing capability of PBMCs on prostate cancer cells

**Fig. 3:**
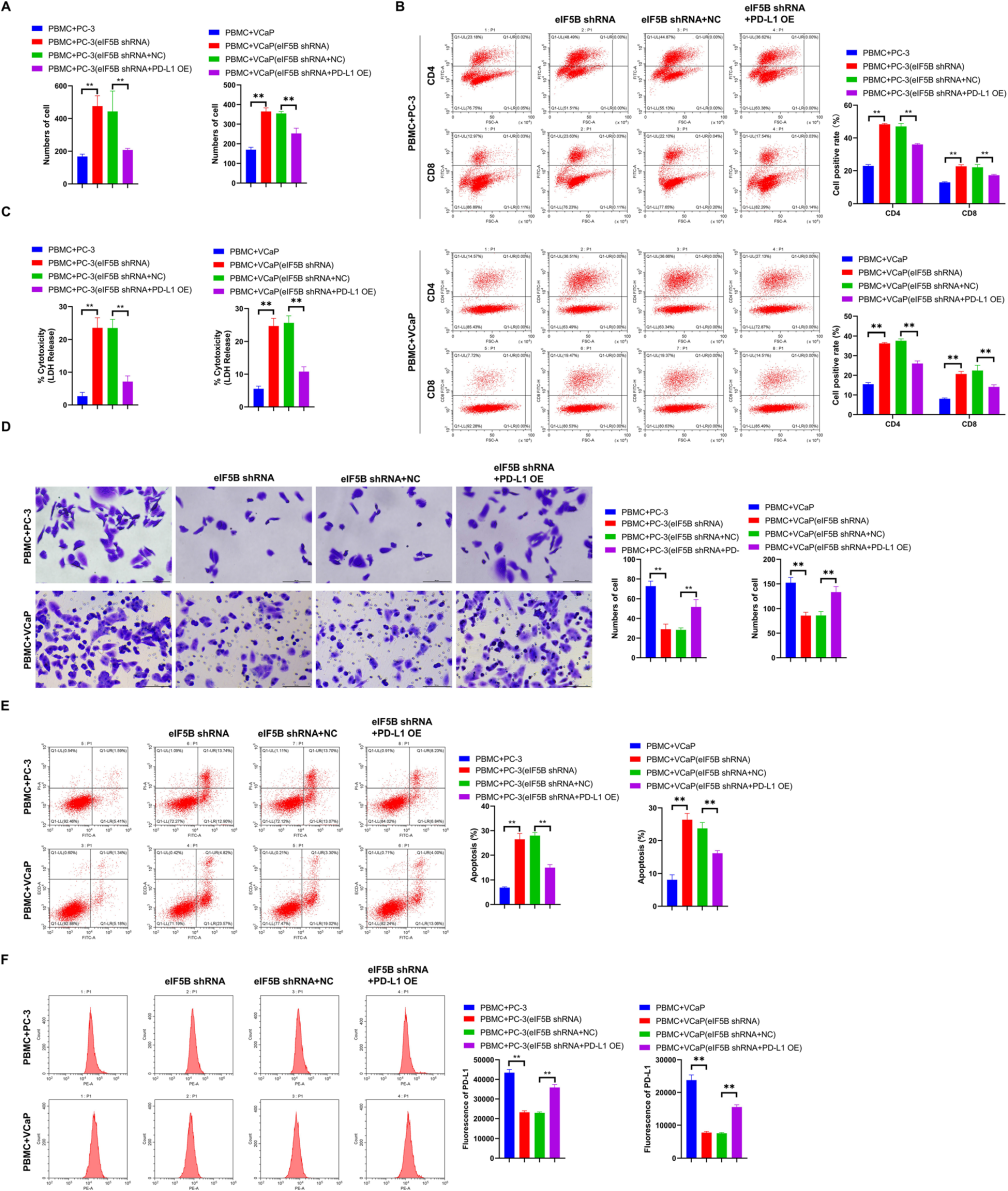
Effects of eIF5B on the killing capability of immune cells. A: The number of lymphocytes was counted under a light microscope; B: The expressions of CD4 + T and CD8 + T cells were detected by flow cytometry; C: The expression of LDH in the supernatant of the co-culture system was detected by an ELISA kit; D The prostate cancer cells stained with crystal violet were observed and counted under a microscope; Apoptosis (E) and PD-L1 expression (F) of prostate cancer cells were detected by flow cytometry; **P < 0.01

To investigate the effect of eIF5B on the killing capability of immune cells, we isolated PBMCs from the peripheral blood of healthy people and co-cultured them with the transfected prostate cancer cells. Our observations revealed that significantly higher numbers of lymphocytes were present in the eIF-5B shRNA groups than the groups without shRNA or those with shRNA and PD-L1 overexpression (*P* < 0.01, Fig. 3A).. Meanwhile, similar conclusions can be drawn with respect to the number of CD4 + and CD8 + cells (*P* < 0.01, Fig. 3B). LDH is an enzyme in the cytoplasm of living cells. Under normal conditions, LDH cannot move across the cell membrane. However, when the target cells are attacked by effector cells, the permeability of the cell membrane changes and LDH can be released into the medium. Therefore, in this study, we used an ELISA kit to observe the LDH amount as an indicator of PBMC-induced cell-killing. The results showed that, the LDH release in eIF5B shRNA groups was significantly higher than that in groups without shRNA or those with shRNA and PD-L1 overexpression (*P* < 0.01, Fig. 3C). Besides, with crystal violet staining and flow cytometry, we also found that the numbers of living cancer cells in eIF5B shRNA groups were significantly lower than those in groups without shRNA or those with shRNA and PD-L1 overexpression. On the other hand, in eIF5B shRNA groups, the proportion of apoptotic cancer cells rose significantly, but PD-L1 expression dropped as well (*P* < 0.01, Fig. 3D-F). These results suggest that interference with eIF5B expression can enhance the killing capability of PBMCs on prostate cancer cells, while PD-L1 overexpression based on the interference of eIF5B expression may weaken such enhancement.

### Effect of Wig1 overexpression on prostate cancer cells

**Fig. 4:**
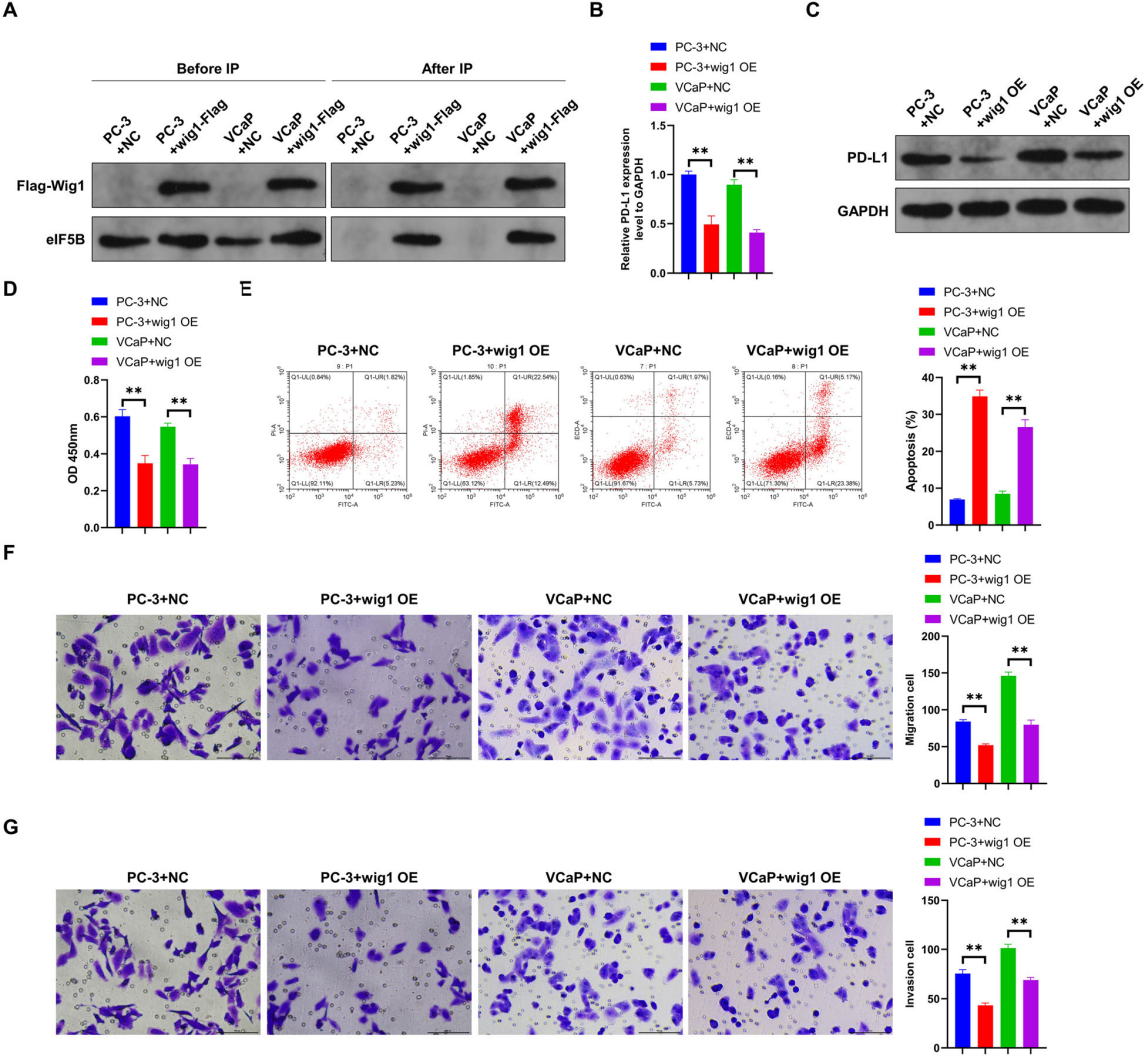
The regulatory mechanism of Wig1 on eIF5B. A: The binding of eIF5B to Wig1 was verified by CoIP assays (full-length blots are presented in Additional file 1); The expressions of PD-L1 in prostate cancer cells were detected by qRT-PCR (B) and Western blot (full-length blots are presented in Additional file 1) (C); Cell proliferation (D), apoptosis (E), cell migration (F), and cell invasion (G) figures of prostate cancer cells; **P < 0.01

CoIP assays were used to verify the regulatory correlations between Wig1 and eIF5B (Fig. 4A). Before IP, Flag-Wig1 was only detected in the Wig1-Flag group, but eIF5B could be detected in both groups. After IP, Flag-Wig1 and eIF5B were not detected in the control group, but both of them were detected in the Wig1-Flag group, suggesting that Wig1 can bind to eIF5B. To further explore the regulatory correlation between Wig1 and PD-L1 in prostate cancer cells, qRT-PCR and Western blot were used to detect the expressions of PD-L1, both its mRNA and protein, in the two prostate cancer cell lines after Wig1 overexpression. The results showed that the cells with Wig1 overexpression revealed significantly lower PD-L1 expression, lower rates of cell proliferation, migration, and invasion, and a higher rate of cell apoptosis (*P* < 0.01, Fig. 4B-G).. These results suggest that the regulatory effects of eIF5B on PD-L1 expressions may be mediated indirectly through Wig1.

### eIF5B silencing inhibits tumor development

**Fig. 5:**
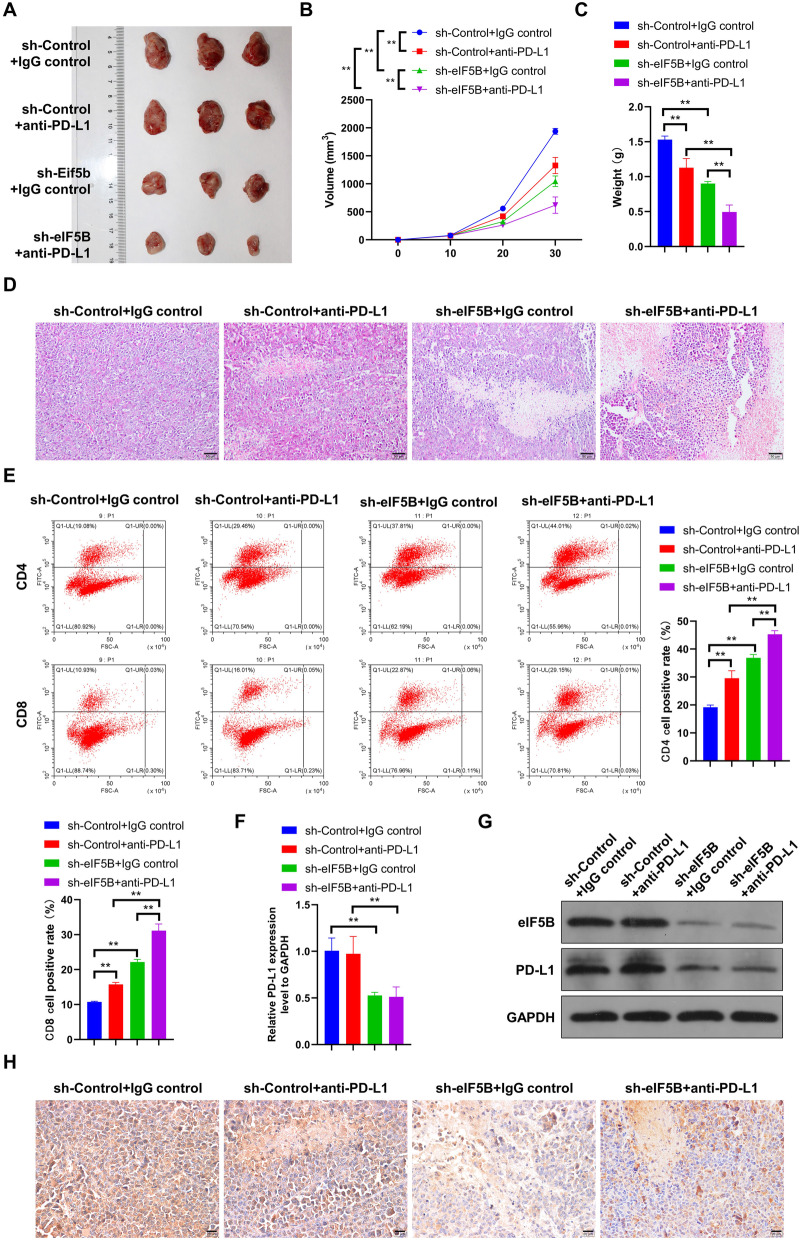
Tumor transplantation in vivo confirmed that interference with eIF5B expression inhibits tumor growth. For each group: (A) tumor tissue photos; (B) tumor tissue volumes; (C) tumor tissue weights; (D) tumor tissue morphology observed by HE staining; (E) CD4+ T and CD8+ T cells in tumor tissues observed by flow cytometry; (F) qRT-PCR observations on the PD-L1 expression; (G) Western blot observations on the PD-L1 expression (full-length blots are presented in Additional file 1); (H) immunohistochemical detection of the eIF5B levels; **P < 0.01

In this study, we constructed sh-eIF5B lentiviruses and transfected them into PC-3 cells to knock down the expression level of eIF5B. PC-3 cells transfected with sh-eIF5B or sh-control were used for tumor transplantation experiments in vivo. When the tumor grew to a volume of 100 mm3, anti-PD-L1 and IgG antibodies were injected into mice. The mice were sacrificed 30 days after tumor transplantation, and the tumors were removed photographed, and recorded for their volumes and weights (Fig. 5A-C). We found that the tumor volumes and weights in the sh-control + IgG control group were the greatest, followed by the sh-control + anti-PD-L1 group, the sh-eIF5B + IgG control group, and the sh-eIF5B + anti-PD-L1 group decreased; in addition, the differences among groups were significant. Subsequently, with HE staining and flow cytometry sessions, we observed that the damages of tumor tissue morphology and the content of CD4 + and CD8 + cells were the smallest in the sh-control + IgG control group, while the two indicators increased following the order of the sh-control + anti-PD-L1 group, the sh-eIF5B + IgG control group, and the sh-eIF5B + anti-PD-L1 group (*P* < 0.01, Fig. 5D and E). These results indicate that PD-L1 antibodies or interference in PD-L1 expression can inhibit tumor growth, destroy tumor tissue morphology, and increase the contents of CD4+ and CD8+ cells, but with eIF5B, such beneficial outcomes can be enhanced. In addition, results from qRT-PCR, Western blot and immunohistochemical analyses showed that compared with the sh-control + IgG control group, PD-L1 and eIF-5B didn’t show much difference in their expression levels in the sh-control + anti-PD-L1 group; however, their expressions were significantly lower in the two groups with sh-eIF5B (Fig. 5F-H). These results suggest that the interference of eIF5B expression, instead of PD-L1 antibodies, could inhibit the expression of eIF5B and PD-L1. Moreover, PD-L1 antibodies could not enhance the changes in the expression of eIF5B and PD-L1 that are induced by the interference of eIF5B expression.

## Discussion

The reasons for tumorigenesis vary in the human body, and many of them are closely related to the autoimmune function of the body. Particularly, immune costimulatory molecules are one of the recent hotspots in immunology research, and PD-1 and its ligand PD-L1are important participants of tumor progression. PD-L1 is widely expressed in tumor cells, and it binds to PD-1 on the surface of T lymphocytes to transmit inhibitory signals to T cells, thereby inactivating T lymphocyte immune reactions [14, 15]. Therefore, inhibition of the PD-1/PD-L1 pathway can enhance T cell function and facilitate tumor cell death, which is a new strategy for tumor therapy.

Translation in cells is a basic step in regulating gene expression, and the abnormal translation may lead to tumors. In eukaryotic cells, mRNA translation is regulated by many eIFs. Micha è l et al. found that eIF4F can regulate PD-L1 expression in human melanoma cells by affecting the translation of signal transducer and activator of transcription 1 (STAT1), which is an important transcription factor [16]. Luis et al. proved that eIF4G1 was highly expressed in NSCLC cells, and its expression level was positively correlated with PD-L1 expression [17]. Specifically, eIF5 contains an unusual amino acid named hypusine that is very important for the proliferation of eukaryotic cells. Its expression has been confirmed to rise in many tumors and is believed to participate in the regulation of cell proliferation and apoptosis [18, 19]. Particularly, eIF5B, a subunit of eIF5, has been found to promote the translation of pro-survival and anti-apoptotic proteins in glioblastoma multiforme cell lines [20] but has never been studied in prostate cancer. In this study, we found compared with normal prostate epithelial cells, the expression of eIF5B and PD-L1 were significantly up-regulated in prostate cancer cells. Interfering the expression of eIF5B could inhibit the proliferation of prostate cancer cells and PD-L1 expression, thereby enhancing the killing capability of PBMCs on prostate cancer cells. Moreover, PD-L1 overexpression based on interference of eIF5B expression can inhibit the effect of eIF5B. In addition, we demonstrated that both the interference with eIF5B expression or the administration of PD-L1 antibodies can inhibit tumor growth in animals. Our results suggest that inhibition of eIF5B can induce strong anti-tumor immune effects by down-regulating PD-L1.

p53 gene is one of the most extensively studied tumor suppressor genes. It is closely related to cell cycle regulations, DNA repair, cell differentiation, and apoptosis [21–23]. Cortez et al. suggested that p53 mutants could intervene in the immune escape of tumors by regulating the expression of PD-L1 [24]. Moreover, p53 has been verified to mutate in prostate cancer [25]. Wig1, a transcriptional target of p53, encodes an unusual zinc finger protein that participates in post-transcriptional gene regulation. Wild-type p53 can up-regulate the expression of Wig1, and Wig1 overexpression can inhibit the growth of tumor cells [26, 27]. In this study, we demonstrated that Wig1 overexpression could inhibit PD-L1 expression and proliferation of prostate cancer cells, which is consistent with the results of Cortez et al. Furthermore, previous studies have shown that Wig1 can bind to eIF5B at the initial stage of translation, resulting in translation inhibition [13]. Such an idea was confirmed in this study. With Wig1 overexpression, PD-L1 dropped and cancer cell apoptosis was promoted, suggesting that eIF5B regulates PD-L1 expression indirectly through Wig1.

## Conclusion

In general, we confirmed that eIF5B can promote the expression of PD-L1 by interacting with Wig1. Interference of eIF5B expression can inhibit the proliferation, migration, invasion, and immunosuppressive response of prostate cancer cells, as well as tumor growth. However, our current study still failed to address several issues. Firstly, the binding of eIF5B and Wig1, which was detected by CoIP assays, may not be direct, and there may be a third intermediate party. Secondly, the regulatory effects of eIF5B on the expression of PD-L1 in prostate cancer cells have only been confirmed at the cellular level, and further verification at the clinical level is needed for greater clinical significance.

## Supplementary Information

**Additional file 1.** The original western blot data of Fig. 1A/2B/4A/4C/5G.

## Data Availability

All data generated or analysed during this study are included in this published article and its additional file.

## Abbreviations

LDH: Lactate dehydrogenase
PBMCs: Peripheral blood mononuclear cells
PD-L1: Programmed death ligand 1
eIFs: Eukaryotic translation initiation factor
Wig-1: Wild-type p53-induced gene 1
FBS: Fetal bovine serum
FITC: V-fluorescein isothiocyanate
PI: Propidium iodide
qRT-PCR: Quantitative real-time PCR
CoIP: Co-immunoprecipitation
STAT1: Signal transducer and activator of transcription 1
ATCC: American Type Culture Collection
