# Supplementary figures and images for: eIF5B regulates the expression of PD-L1 in prostate cancer cells by interacting with Wig1
Source: BMC Cancer. 2021 Sep 15;21:1022. doi: 10.1186/s12885-021-08749-w (PMC8442339; doi:10.1186/s12885-021-08749-w)

Figure 1A:


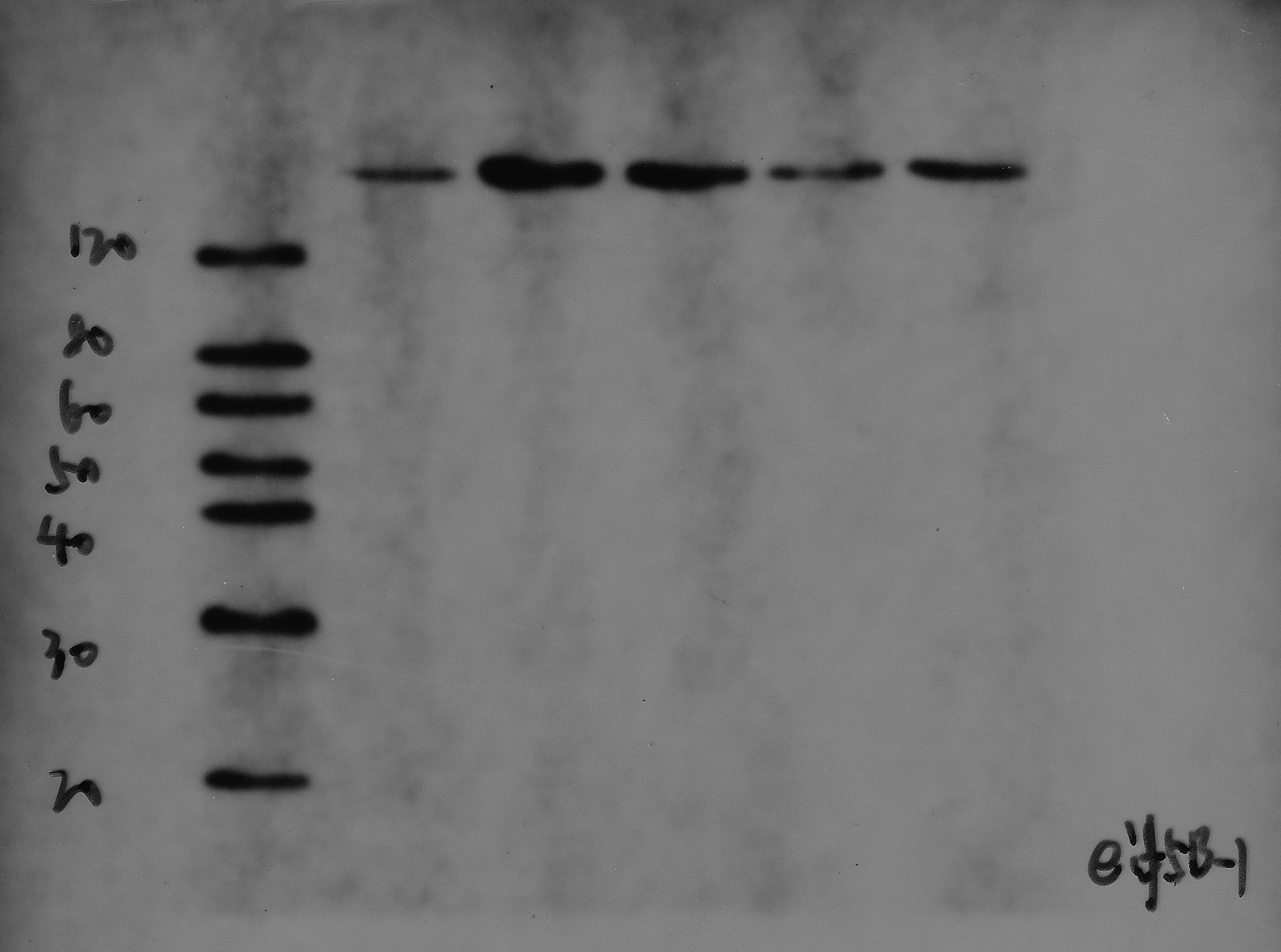


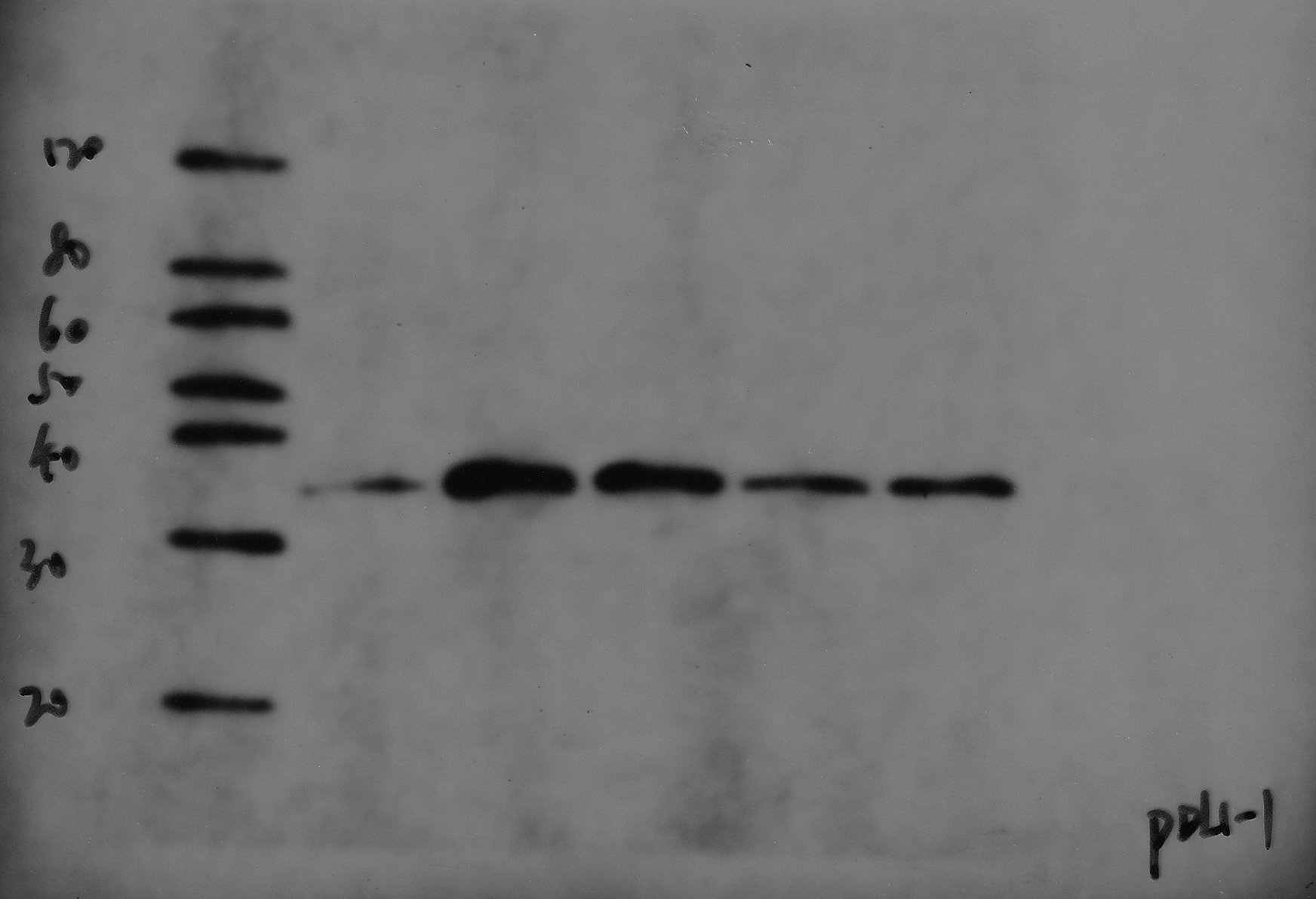


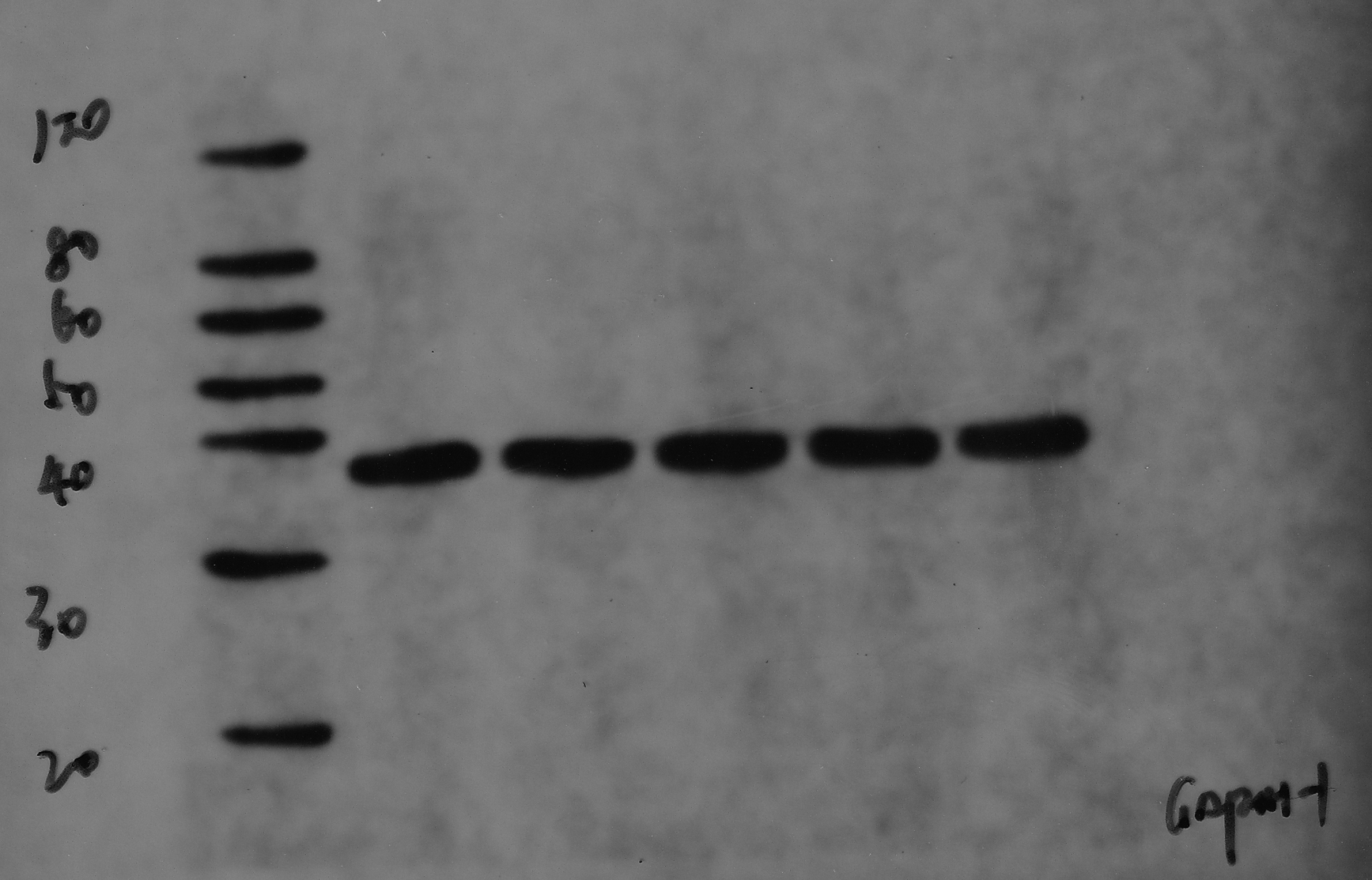


Figure 2B:


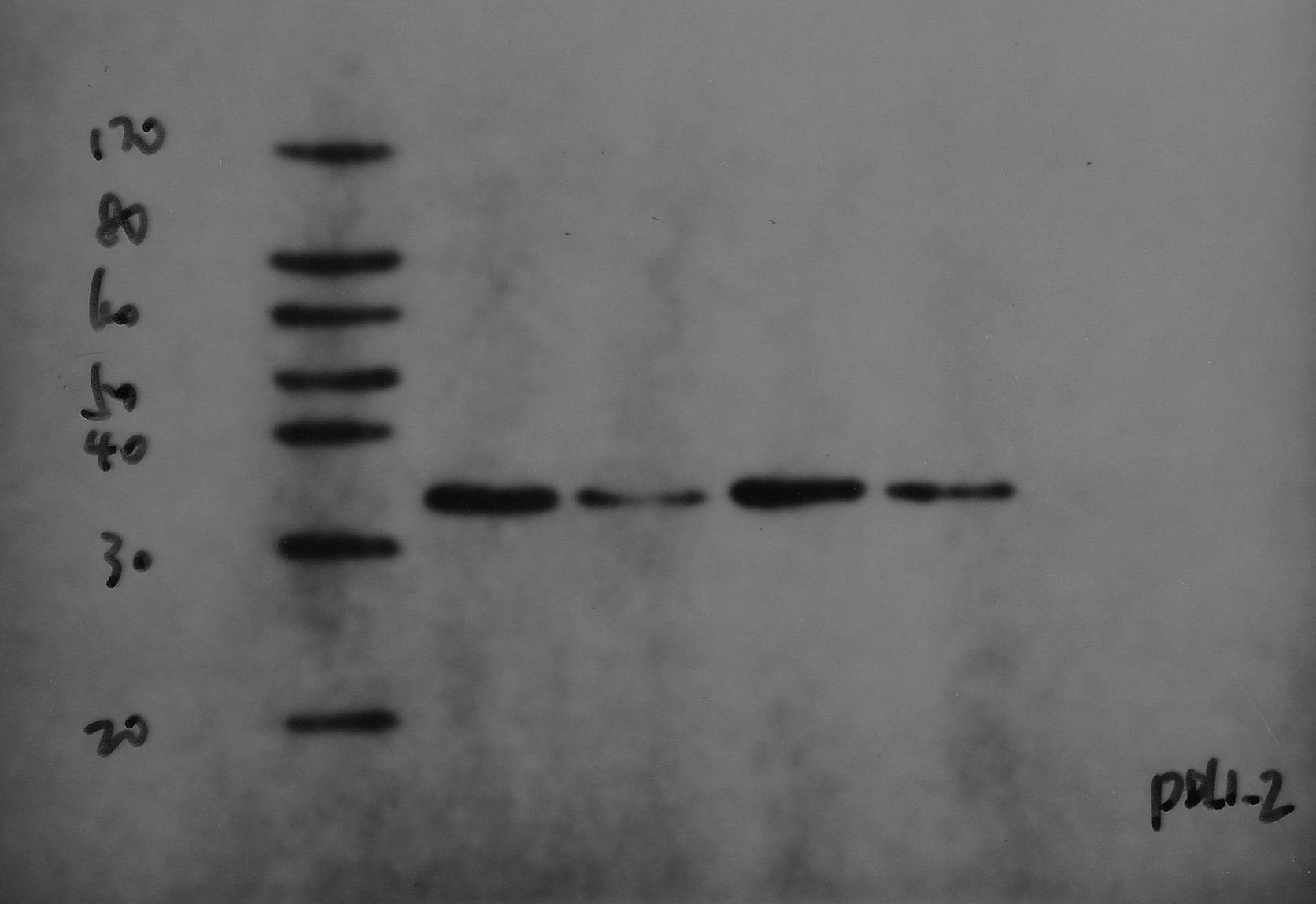


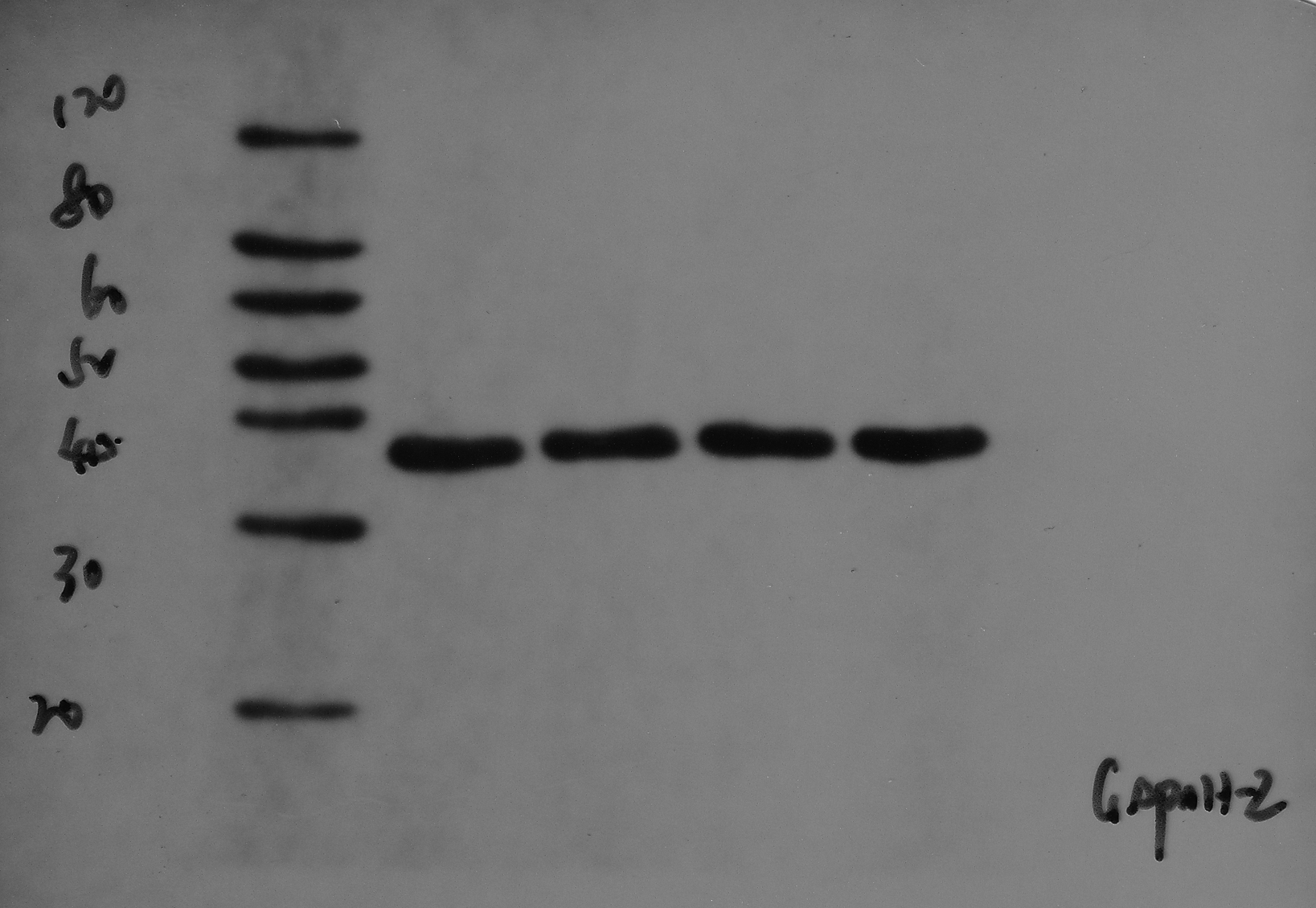


Figure 4A:


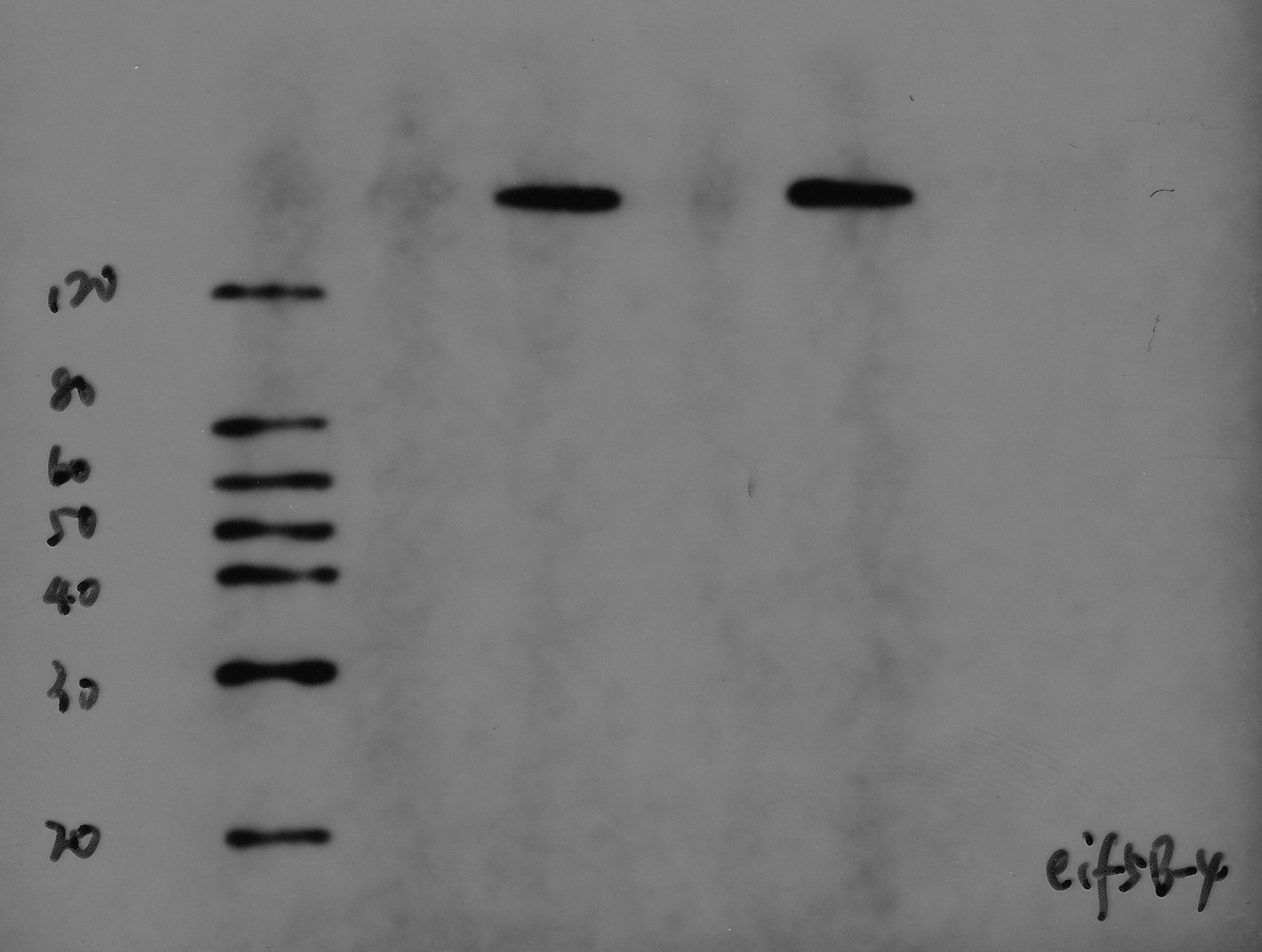


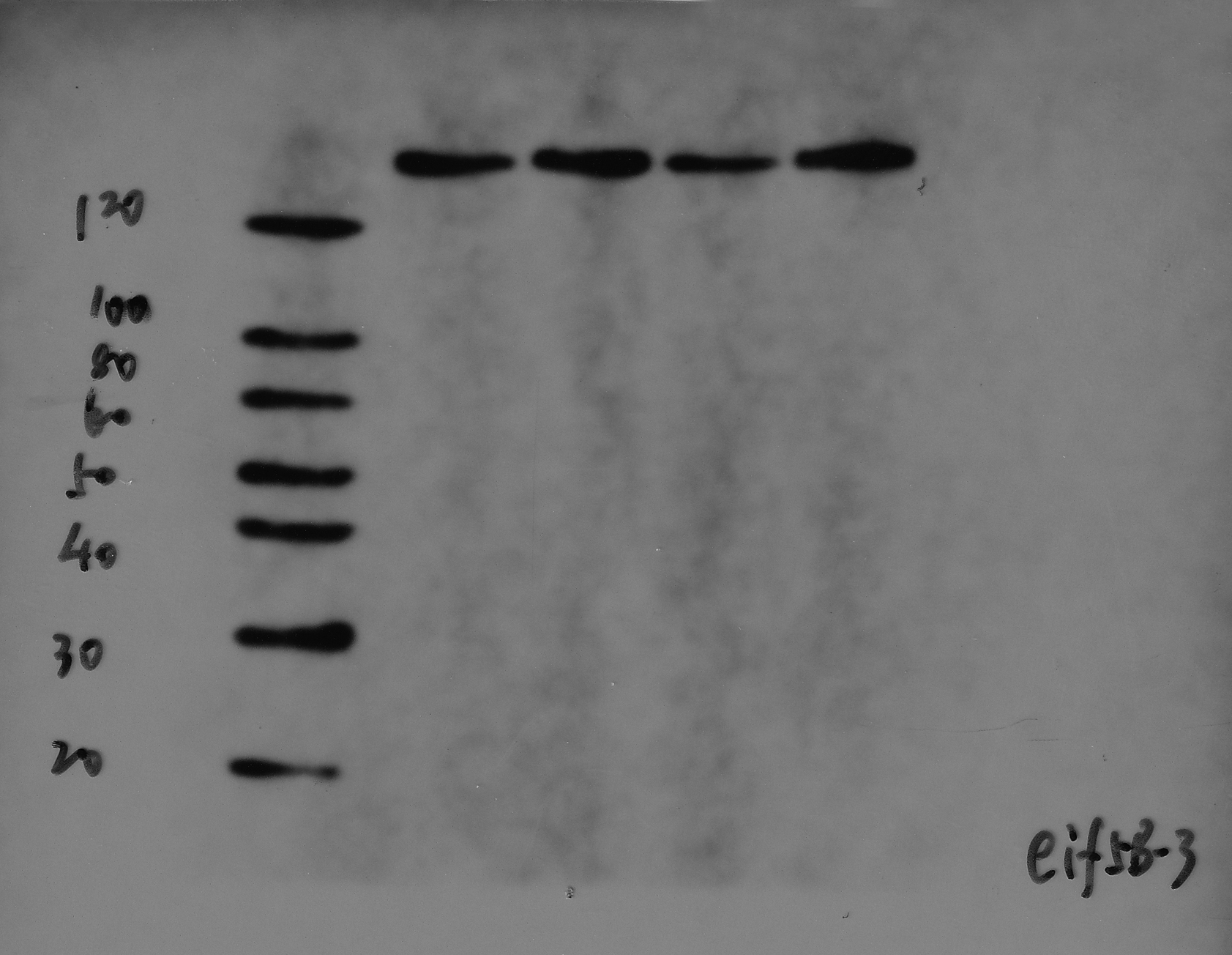


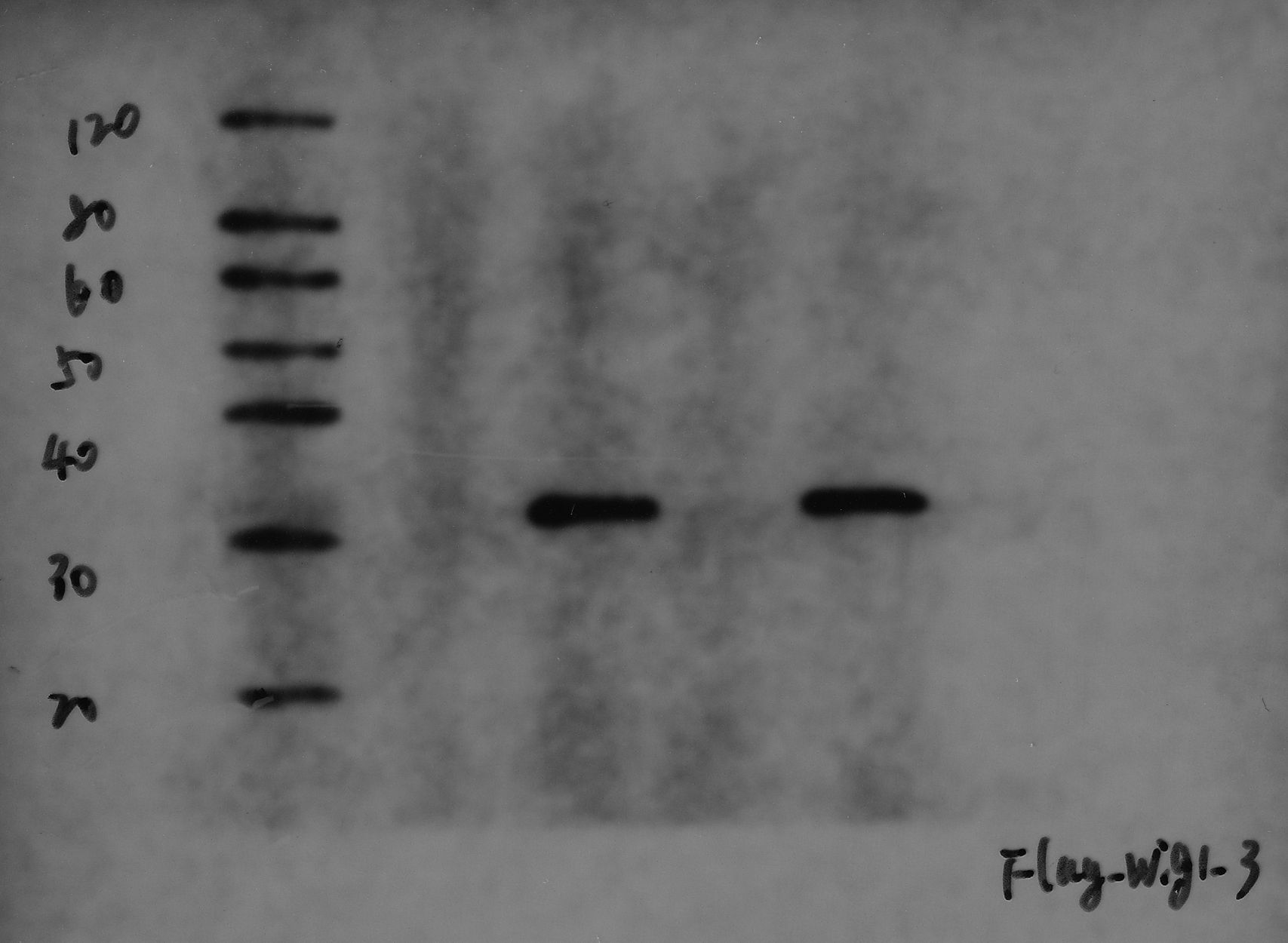


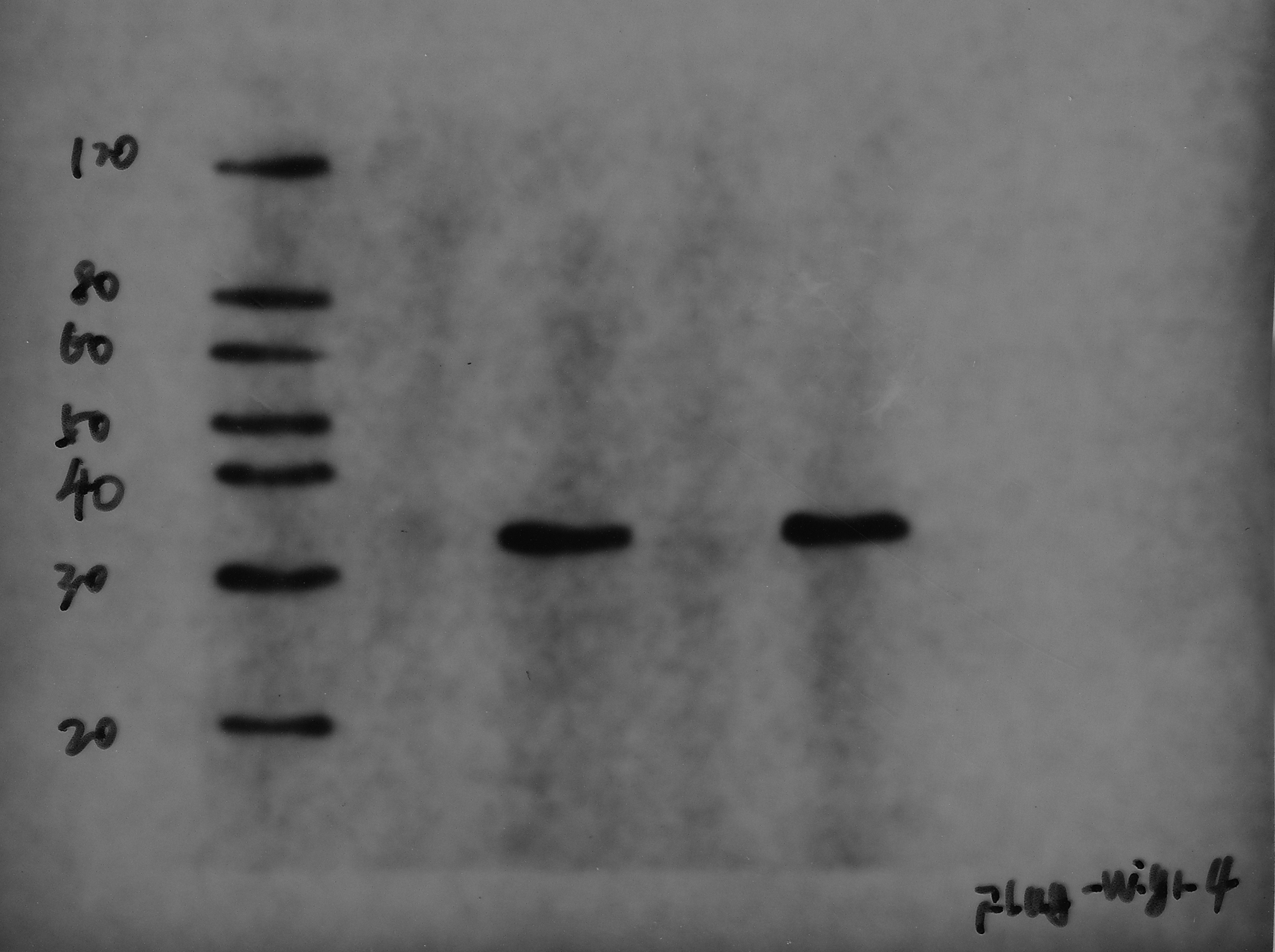


Figure 4C:


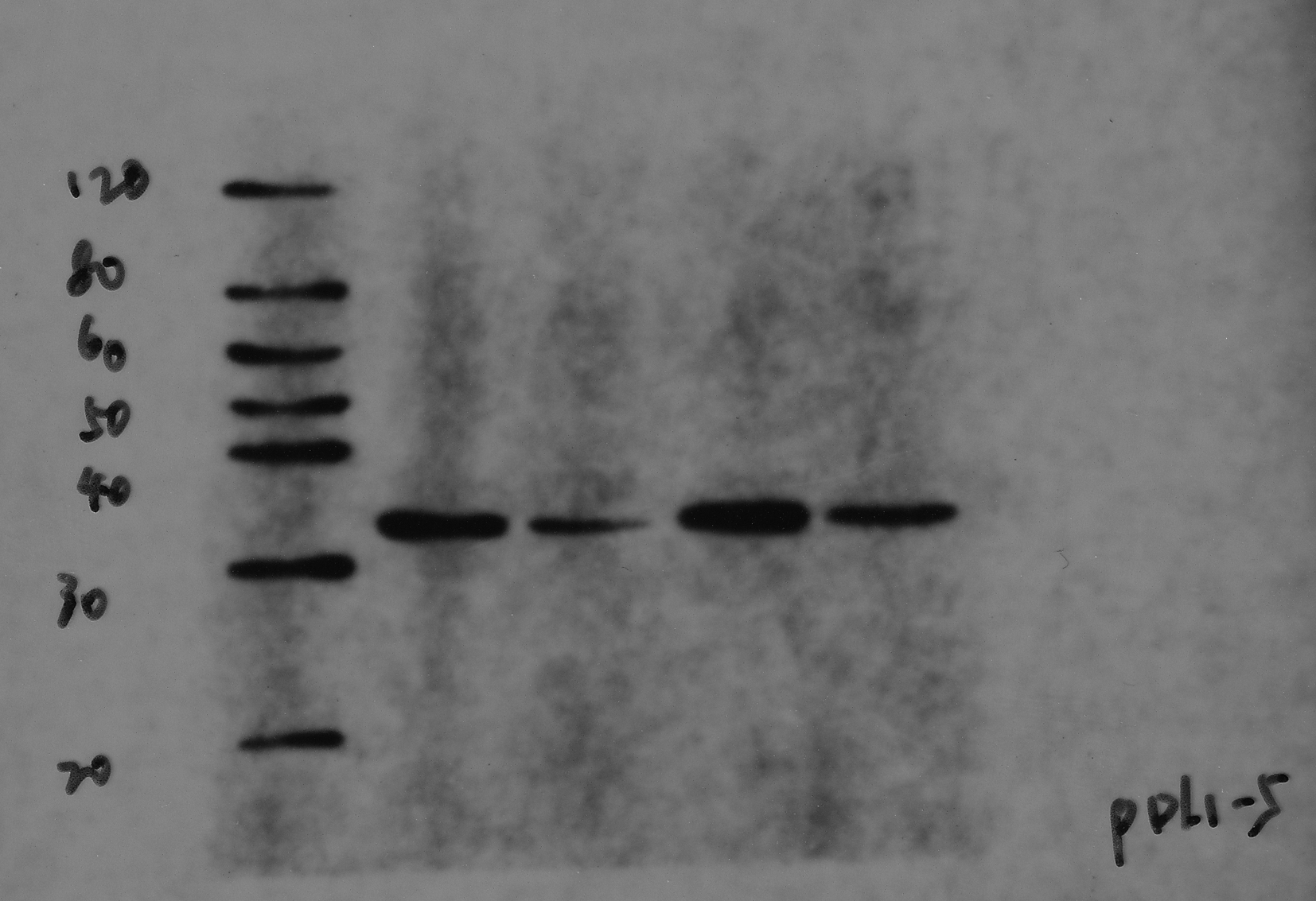


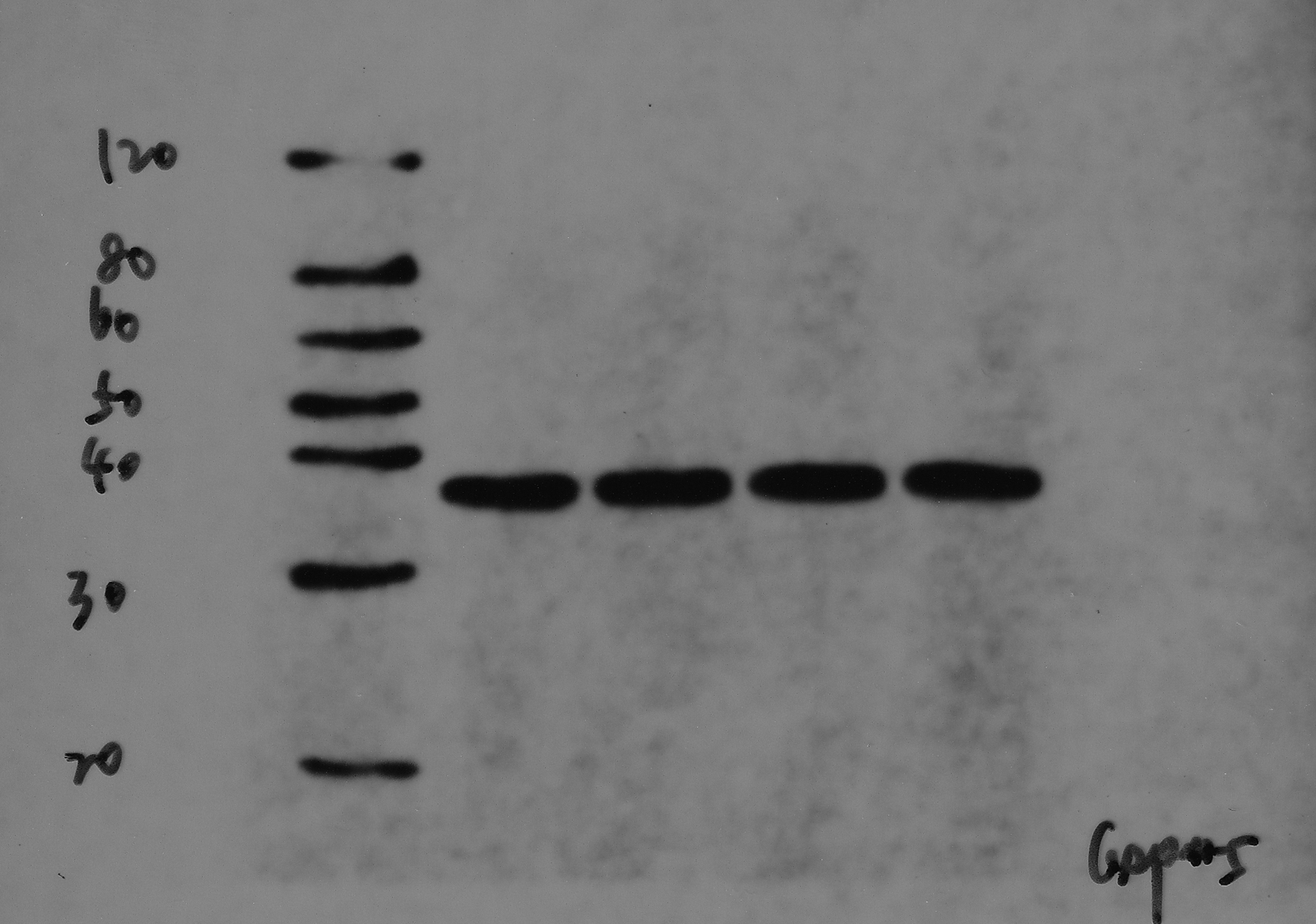


Figure 5G:


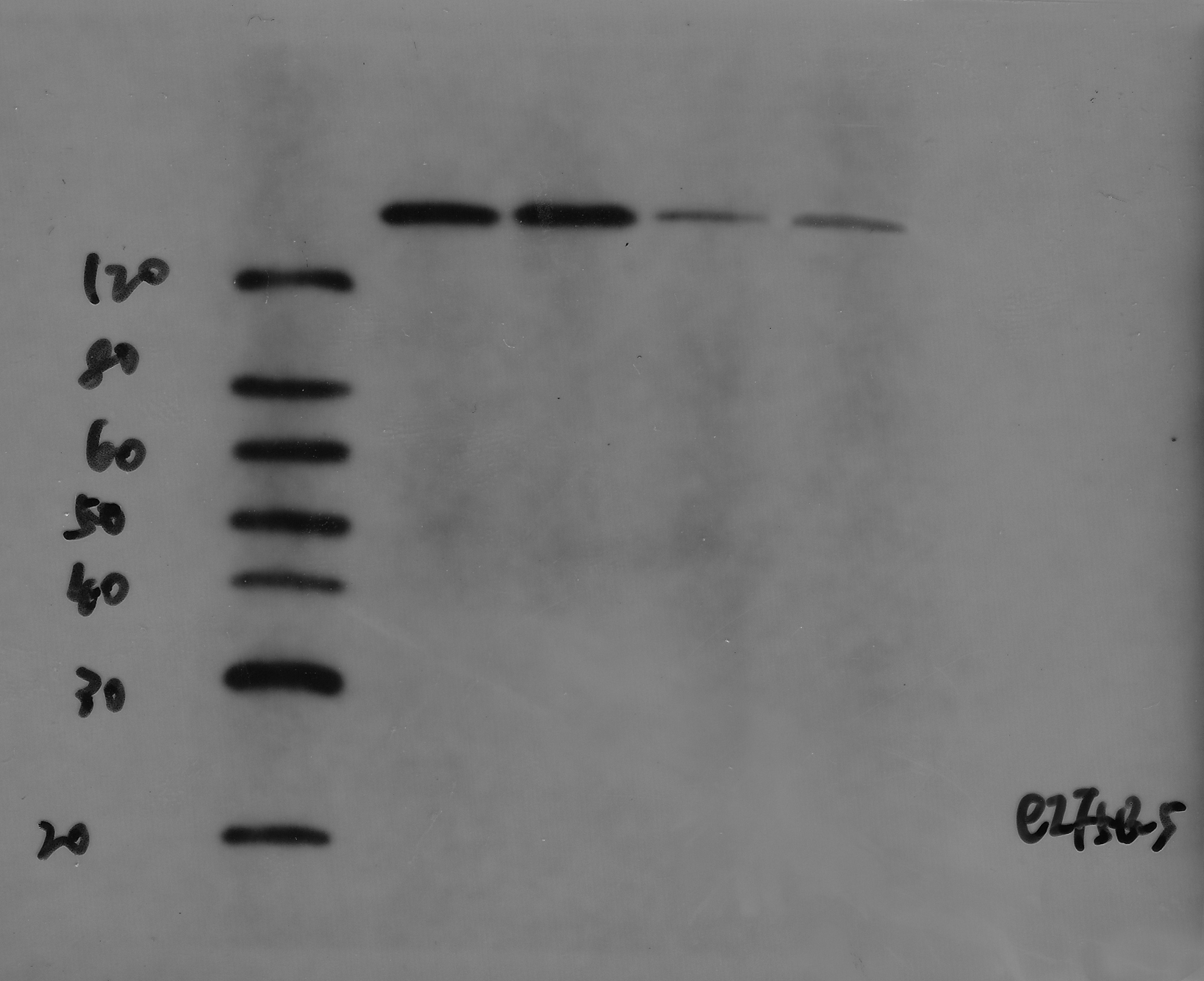


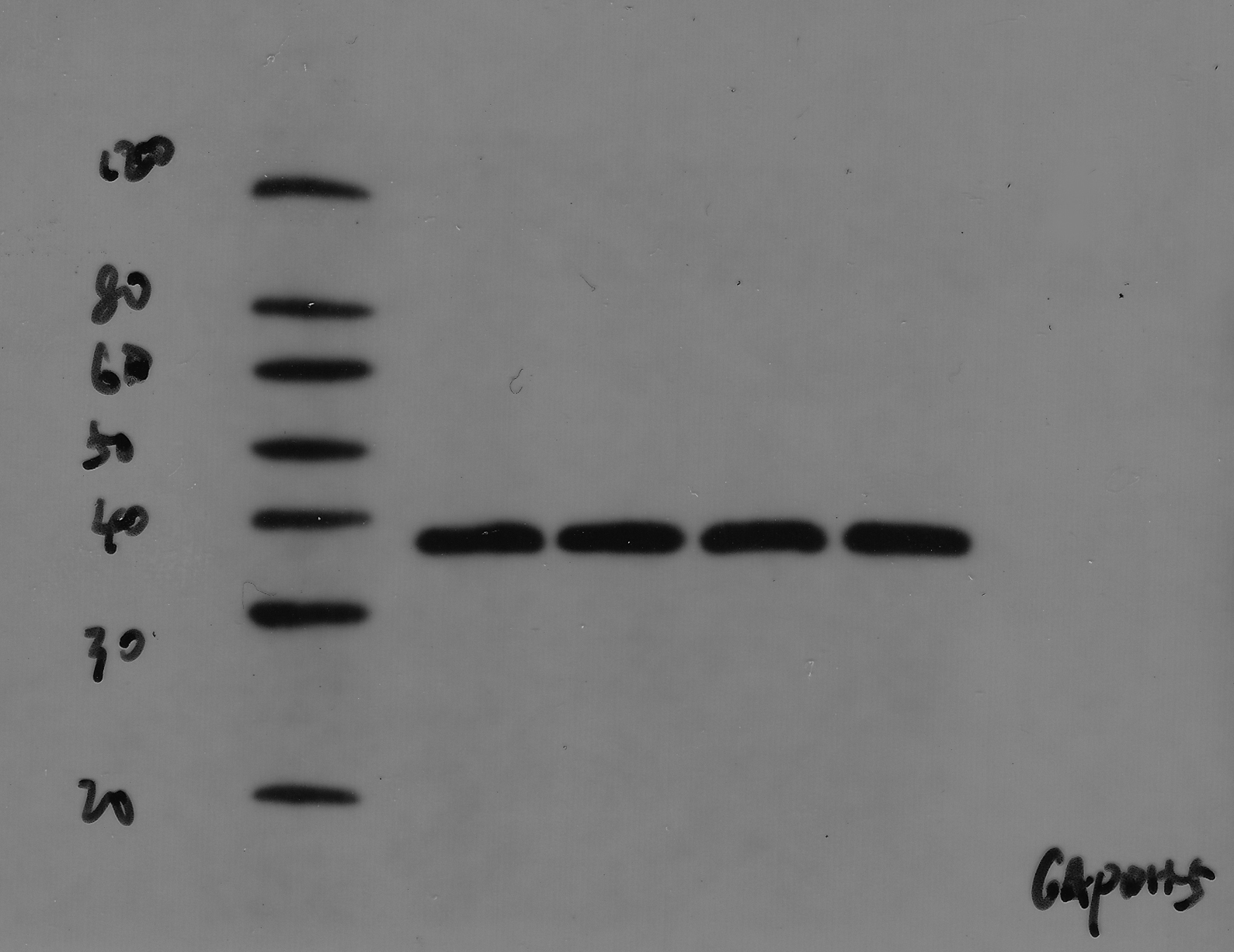


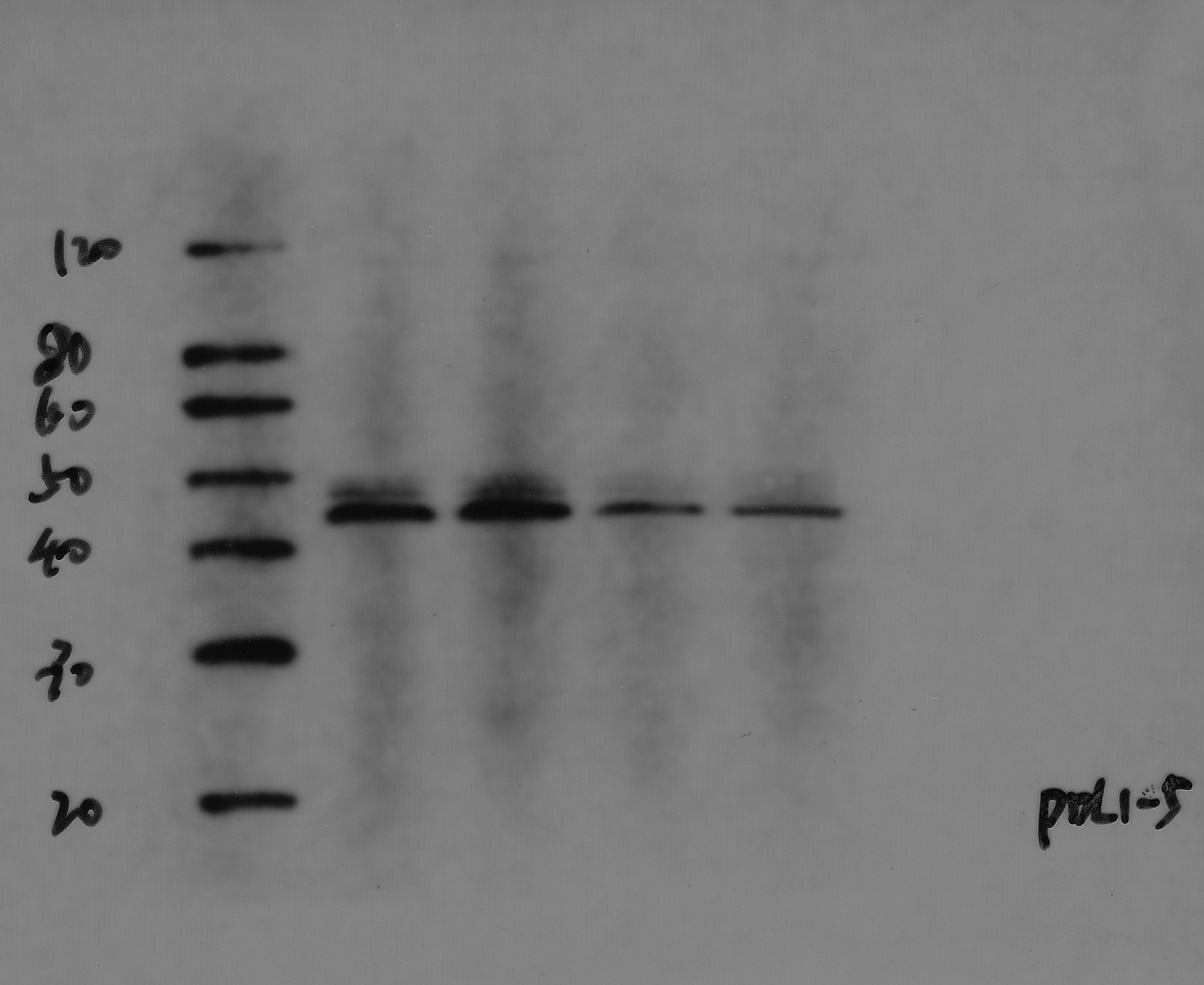

Supplement: Supplementary file 1 — Additional file 1. The original western blot data of Fig. 1A/2B/4A/4C/5G. [file 12885_2021_8749_MOESM1_ESM.doc]
